# *Vibrio Fluvialis*: An Unusual Enteric Pathogen of Increasing Public Health Concern

**DOI:** 10.3390/ijerph7103628

**Published:** 2010-10-12

**Authors:** Etinosa O. Igbinosa, Anthony I. Okoh

**Affiliations:** Applied and Environmental Microbiology Research Group (AEMREG), Department of Biochemistry and Microbiology, University of Fort Hare, P/Bag X1314, Alice 5700, South Africa; E-Mail: eigbinosa@gmail.com

**Keywords:** *vibrio fluvialis*, environmental/public health impact, epidemiology

## Abstract

In developing countries, the fraction of treated wastewater effluents being discharged into watersheds have increased over the period of time, which have led to the deteriorations of the qualities of major rivers in developing nations. Consequently, high densities of disease causing bacteria in the watersheds are regularly reported including incidences of emerging *Vibrio fluvialis. Vibrio fluvialis* infection remains among those infectious diseases posing a potentially serious threat to public health. This paper addresses the epidemiology of this pathogen; pathogenesis of its disease; and its clinical manifestations in humans.

## 1. Introduction

*Vibrio fluvialis* is a halophilic Gram-negative bacterium. It has a straight to slightly curved rod cell morphology that is motile by means of polar flagella. It is a sodium chloride-requiring, oxidase-positive, nitrate-positive organism that ferments D-glucose and other carbohydrates with the production of acid and gas; has 50% guanine-plus-cytosine in its DNA [1]; and is isolated from water, animal feces, human feces, sewage, and seafood product. *V. fluvialis* is an important cause of cholera-like bloody diarrhoea and causes wound infection with primary septicemia in immunocomprised individuals from developed to underdeveloped countries, especially in regions with poor sanitation.

In 1977, Furniss *et al.* [2] reported the isolation of a new group of bacteria, designated, group F, in Bahrain from humans suffering from severe diarrhoea. The newly described bacteria possessed phenotypic properties intermediate between those of *Vibrio* spp. and *Aeromonas* spp. and were associated with gastroenteritis. Subsequently, results of a numerical taxonomy study of 154 strains of *Vibrio* spp., *Aeromonas* spp., and group F organisms were reported by Lee *et al*. [1]. Group F strains were found to cluster within a single group, with two sub-clusters separable on the basis of gas production from glucose. All strains isolated from diarrhoeal patients were found to be anaerogenic, whereas those from the environment included both aerogenic and anaerogenic strains. The authors concluded that group F organisms are more closely related to *Vibrio* spp. than to *Aeromonas* spp. Group F organisms have been reported to be widely distributed in the marine and estuarine environment around Britain [1].

The distribution of *V. fluvialis* is a global phenomenon [3] and this organism is not only isolated from human diarrhoeal cases as reported by previous authors [4,5] but also from aquatic environments [6–8]. We recently reported *V. fluvialis* from a treated wastewater effluent system in South Africa [9]. Also, there are reports of food poisoning caused by this organism [10], especially due to consumption of raw shellfish [11]. The pathogen has also been found in association with extra-intestinal infections [12,13], and in general, the clinical indication of the disease includes mild to moderate dehydration, vomiting, fever, abdominal pain and diarrhoea [6].

Given its public health implication, *V. fluvialis* has been the subject of intensive study for the last two decades, since it was isolated in 1975 and described in 1977 by Furniss and his group. Yet many questions remain unanswered about its microbiological characteristics, virulence-engendering factors, and possible methods of attenuation. Since *V. fluvialis* enteritis is reported infrequently, the epidemiology of this infection is not adequately understood. There is very little information available on the virulence factors associated with its infections and less information on the mechanism of pathogenicity of the organism. In our laboratory, microbial water/wastewater quality studies are in progress and directed at assessing the hazards implications of this organism in the aquatic milieu. The organism has been isolated from treated final effluent from wastewater treatment plants in our recently conducted study both at the rural, sub-urban and urban communities of the Eastern Cape Province of South Africa [9]. In view of our recent findings on *V. fluvialis*, this review addresses the epidemiology of this pathogen; pathogenesis of its disease; and its clinical manifestations in humans as an emerging pathogen of increasing public health significance.

## 2. Classification of *V. fluvialis* Strains

As earlier highlighted *V. fluvialis* were first described by Furniss *et al.* [2]. The organisms, designated group F, were isolated in 1975 from a patient with diarrhoea in Bahrain, from patients with diarrhoea in Bangladesh, and from shellfish and estuarine waters in England. Group F required salt and had a number of properties compatible with or midway between those of vibrios and aeromonads. In a numerical taxonomic study, Lee and co-workers showed that six group F strains were a distinct phenon that probably represented a new species [14], and that the group contained two subgroups on the basis of gas production during fermentation of glucose [1]. Huq *et al.* [4] studied a large number of strains associated with an outbreak of diarrhoea in Bangladesh as well as strains isolated from patients with diarrhoea in Indonesia; strains from sewage in Brazil; and strains from the United States of America that had been called group EF-6 in the Special Bacterial Reference Activity at the Centers for Disease Control. By both phenotypic tests and DNA relatedness, they found that the organism was closer to the genus *Vibrio* than to the genus *Aeromonas*. All their strains produced no gas from the fermentation of glucose (were anaerogenic) and formed a single DNA relatedness group based on GC ratio and DNA-DNA hybridization [15]. Thus, the EF-6 group appeared to be identical to group F. Group F strains isolated from several parts of the world were compared phenotypically and genetically by Seidler *et al.* [6]. They confirmed and extended the observation that group F was more closely related to *Vibrio* than to *Aeromonas*. They further showed that the aerogenic group F strains were in a different DNA relatedness group from the anaerogenic strains, and they recommended that the two biogroups be considered as two separate species within the genus *Vibrio*. Lee *et al.* [7] proposed the name *V. fluvialis*, which included both aerogenic and anaerogenic strains of group F and the synonymous group EF-6. An anaerogenic strains was chosen as the type strain of *V. fluvialis*. These authors noted that both aerogenic and anaerogenic strains of *V. fluvialis* were found in the environment but that only anaerogenic strains had been isolated from humans with diarrhoea [7].

## 3. Sources and Routes of *Vibrio fluvialis* Infection Transmission to Human

There is a complex interaction of environmental and behavioural factors of both animals and humans, which facilitate the spread of *V. fluvialis* infections. Poor sanitation and hygiene conditions as well as lack of or little environmental awareness among people is considered the major cause of source water contamination. Typical examples include agricultural practices that involve usage of sewage water and/or cattle manure on farms. Another practice is uncontrolled wastewater effluents discharges into waterbodies, which serve as raw water sources to municipal water treatment systems; unhygienic/unconventional sanitation practices in velds; and grazing of cattle next to catchment areas [16]. Enteric infections cause considerable morbidity and mortality worldwide, especially among children in developing countries. *V. fluvialis* infections are common in areas that have high levels of fecal contaminated water, food supplies and consumption of raw seafood or contaminated seafood products [16], as well as by person-to-person contact [17]. Infection rates are highest where general standards of living, water supply, and sanitary conditions are low or inadequate, although microbial contamination of water remains the largest and most immediate health hazard, with surface water quality been subjected to frequent dramatic changes in microbial quality as a result of the variety of activities on the watershed [18,19]. These changes could be caused by discharges of municipal raw waters or treated effluent at a specific point-source into the receiving waters [20].

Okoh *et al.* [21] reported on the qualities of wastewater effluents of some crude oil flow stations in the Niger delta which are channeled into saver pits prior to discharge into the environment including waterbodies such as streams or rivers, and suggested that such saver pits could be potential sources of pathogens in the watershed. Also, in rural and sub-urban communities of most developing nations, the reuse of sewage and wastewater is often the only source of water for irrigation in these areas, and eating fruits and vegetables that have been irrigated with inadequately treated wastewater is a most likely route of contracting *V. fluvialis* infection [9,17,19,20]. The practice of direct discharge of effluents into receiving waterbodies is of major concern as it could result amongst other things in the substantial increase in organic load and consequently depletion of the dissolved oxygen content of the receiving waterbodies [19–21].

There are ongoing debates about the origins of emerging *V. fluvialis* pathogen, but most attribute at least some causation to global changes impacting on the social and natural environments. There is a speculation that *V. fluvialis* strains evolved from highly polluted waters with intense population pressure in large urban concentrations; intensification of farming to feed growing populations; and widespread poverty and inequalities in the region. Tons of pesticides, millions of gallons of industrial waste and raw sewage, and millions of tons of chemical fertilizers are dumped daily into local rivers flowing into the bay. It was previously thought that pollutants dumped into the sea would quickly degrade as it sunk downwards, but there is growing evidence that microbes and other biological material can survive in suspended animation in the ocean depths, capable of resurfacing via the food chain or ocean currents [22]. This human-induced degradation of the local environment is also leading, it is believed, to widespread changes in the coastal ecology [23].

An alternative environmentally-focused theory argues that patterns of cholera-like diarrhoea epidemics in South Asia can be linked to patterns of global climates changes. Human-induced climates changes may be creating favourable conditions such as water, temperature, nutrient concentration and plankton production, for the growth and reproduction of the bacterium [23].

## 4. Epidemiology

*Vibrio fluvialis* is considered to be one of the foodborne pathogenic bacteria and has been implicated in outbreaks and sporadic cases of diarrhoea [24]. Existing naturally in warm, salty, and brackish water, *V. fluvialis* survive in temperature between 9 °C and 31 °C, but thrives when water temperature rises above 18 °C. *V. fluvialis* infections demonstrate a seasonal pattern with the majority of clinical illnesses when temperature and salinity factors are most favourable to the bacteria’s proliferation [9]. In Bahrain, *V. fluvialis* was identified for the first time as group F *Vibrio* in 1975 in a patient with diarrhoea [2], and it was later named *V. fluvialis* [7]. Between October 1976 and November 1977, the largest outbreak of *V. fluvialis* infection was reported in Bangladesh [4]. The outbreak involved more than 500 patients, with 50% of them being young children.

In the United States, *V. fluvialis* has been associated with enterocolitis in infants [25], and has also accounted for 10% of clinical cases in a survey of *Vibrio* infections along the Gulf Coast [11]. It was also detected in the stools of persons with diarrhoea in Jordan, Yugoslavia [7], and Bahrain [2]. In the United States, the organism was isolated from a wound of a patient in Hawaii; from water and sediment in the New York Bay [6]; from shellfish in Louisiana; and from water and shellfish in Pacific Northwest estuaries [26]. Recently it was reported to be associated with acute diarrhoea in Indonesia [27]. Currently, *V. fluvialis* has an infectious importance because its clinical symptoms of gastroenteritis are very similar to that of *V. cholerae.* The matter became more serious after the recent characterization of an enterotoxigenic El Tor-like hemolysin in *V. fluvialis*, which represents one of the virulence factors of *V. cholerae* [28]. *V. fluvialis* have been reported as causing necrotising fasciitis and septicaemia in the Gulf of Mexico and Southeast Asia, associated with minor trauma and exposure to fish, raw oyster, shellfish, crabs or seawater, especially in the summer months [29–31].

## 5. Survival of *Vibrio fluvialis* and Other Vibrios in Environments

*Vibrio* spp. are, in most cases, found as free planktonic bacteria in the environment but in complex multispecies biofilm structures attached to various biotic and abiotic surfaces [9,42,43]. They are also often found attached to chitinaeous exoskeleton of zooplantkton [42,43]. Biofilms contribute to the survival of bacterial communities by promoting interspecies metabolic and genetic cooperation as well as protection against diverse environmental stresses, such as starvation and predation [44–46].

Usually, lack of nutrient is the most common environmental stress which microorganisms routinely encounter in natural ecosystems. However, it was found that *Vibrio* spp. can survive for a long time during starvation by sequential changes in cell physiology and gradual changes in morphology [47–49]. Moreover, it was reported that some species develop the so-called viable but non culturable (VBNC) state in response to certain stress conditions [50–52], and it has been proposed that the VBNC state is an adaptative strategy of microorganisms against stress from which cells may be able to recover once optimal conditions are restored [53–55]. VBNC state was described for many *Vibrio* species [*V. anguillarum*, *V. campbellii*, *V. cholerae*, *V. fischeri*, *V. harveyi*, *V. mimicus*, *V. natriegens*, *V. parahaemolyticus*, *V. proteolyticus*, *V. vulnificus*] [56]. Amel *et al.* [57] studied survival of *V. fluvialis* in seawater under starved condition and found that *V. fluvialis* have developed strategies that allow it to survive in seawater in the absence of nutrients and outside its natural host during a long period of time. Under these conditions, this bacterium maintains its virulence factors.

A long-term starvation survival has been described for both *V. anguillarum* and *V. salmonicida* that are able to survive for more than 60 weeks in seawater at a temperature of 6–8 °C [58]. Bacterial survival is further enhanced when surfaces are available for their attachment or when they are located in sediments [59]. *V. salmonicida* was isolated in the sediment of a fish farm more than 18 months after an outbreak of vibriosis [60].

Many enzymes, which can metabolize aquatic substrates and contribute to bacterial survival in the environment, have been identified in several *Vibrio* spp. Agarases are enzymes that degrade agar, a compound found in the cell walls of algae, releasing a metabolisable product that is used as an energy source [61,62]. Chitinases degrade chitin, a homopolymer of N-acetylglucosamine, which is the major component of the cell walls of many organisms such as fungi, crustaceans and insects. Chitin is the largest pool of amino sugars in the oceans and the ability to degrade it confers an important survival advantage [63]. Chitinase activity has been detected notably in *V. anguillarum*, *V. furnissi* and *V. cholerae* and more than 10 enzymes with chitinase activity are produced by *V. harveyi* [64–66]. *In vitro*, *V. cholerae* uses chitin as a sole carbon source for growth [67], providing to the bacterium the potential to use a readily available nutrient source in aquatic environments and to colonise ubiquitous marine environments. These long-term starvation strategies in seawater or attachement to surfaces in the aquatic environment indicate that most of the pathogenic vibrios are endemic species in the marine environment. They can survive during a relative long period in various milieus and re-infect their respective marine host when the conditions become favourable [53].

## 6. Clinical Manifestations

*Vibrio fluvialis-*related illness is characterized by gastroenteritis, nausea, loss of appetite, vomiting, watery bloody diarrhoea with abdominal cramps or significant fever. Moderate to severe dehydration, hypokalemia, metabolic acidosis, and occasionally, hypovolemic shock can occur in 4 to 12 hours if fluid losses are not replaced. Stools are colourless, with small flecks of mucus and contain high concentrations of sodium, potassium, chloride, and bicarbonate.

In the wound infection (cellulitis) that is caused by direct inoculation of bacteria into the skin or exposure of a wound to contaminated water [37], the bacterium (and its associated toxins) rapidly cause local tissue necrosis associated with hemorrhagic bullae and erosions [37]. Cellulitis may occur when an abrade area of skin is inoculated through bathing in marine waters where *V. fluvialis* thrives, or through exposure to liquid from harvested raw seafood [8,37]. This type of exposure typically occurs while sucking or handling raw oysters. Since the organism causes obliterating vasculitis and vascular necrosis, therapeutic levels of antibiotics may not reach the organism and rapid amputation may be necessary to prevent progression.

The primary septicaemia syndrome consists of high fever and chills, often with vomiting, diarrhoea, abdominal pain and extremities pain [8] with no apparent focus of infection. Major diagnostic clues for *V. fluvialis* sepsis syndrome are heamorrhagic bullae which can be seen both in sepsis and cellulitis [8]. It is believed that the bacteria most likely enter the circulation through the intestine [8].

A number of host factors predisposed patients to severe infection with *V. fluvialis.* Known adverse host factors include liver disease (especially alcoholic cirrhosis), immunocomprised states such as HIV/AIDS, iron overload (e.g., hemochromatosis), and diabetes mellitus [8].

## 7. Pathogenesis of Disease

The symptoms of enteric disease attributed to *V. fluvialis* are similar to those caused by *Vibrio cholera* [4]. Patients typically have watery diarrhoea with vomiting, abdominal pain, moderate to severe dehydration and often fever. A notable difference from cholera is the frequent occurrence of bloody stools in infections due to *V. fluvialis* [4]. From the enzyme-linked immunosorbent assay, Chikahira and Hamada [68] and Wall *et al.* [69] have reported that several *V. fluvialis* strains isolated from environmental and human sources produced an enterotoxin which is immunologically indistinguishable from cholera toxin (CT).

*V. fluvialis* produces several toxins that may be important in pathogenesis including an enterotoxin-like substance, lipase, protease, cytotoxin, and hemolysin [28,33,34]. Baffone *et al.* [35] reported that *V. fluvialis* has weak adhesiveness and no bacterial cytotoxicity, but Wong *et al.* [34] found it had strong haemolytic and proteolytic activity. Two cases of fatal infection due to *V. fluvialis* have been reported [32]. It accounted for 10% of *Vibrio* gastroenteritis cases in a US survey [36]. Unlike other *Vibrio* spp., which have commonly been reported to cause extra-intestinal infections, *V. fluvialis* is uniquely associated with gastroenteritis, with only rare reports of extra-intestinal infections such as hemorrhagic cellulitis with cerebritis, bacteremia, and peritonitis [37–41].

### Virulence Factors

In spite of a significant volume of published research efforts to elucidate virulence factors of *V. fluvialis* that are responsible for the notable disease process, very little definitive information have been achieved. Several virulence factors have been identified in *V. fluvialis*, but the majority of them are only partially characterized and their precise role in virulence remains to be known.

Endotoxin activity of *V. fluvialis* has been demonstrated *in vitro* using Chinese hamster ovary (CHO) cells. Lockwood *et al.* [70] reported that at least four biologically active substances could be found in culture supernatants of *V. fluvialis* strain 5489. CHO cell elongation factor, CHO cell killing factor (CKF), and cytolysin active against rabbit erythrocytes were identified when the bacterium was grown without lincomycin. Finally, CHO cell rounding toxin, which is known to be a protease, was found. CKF was internalized and cell death was induced by disruption of cellular function [69]. These four active substances were heat-labile and each crude concentrate caused fluid accumulation in the small intestines of infant mice. Of many virulence factors produced from *V. fluvialis*, hemolysin was thought to be of most importance.

Hemolysin has been known to be an important virulence factor in the pathogenic processes of many clinical microorganisms, causing hemorrhagic septicemia and diarrhoea [71,72]. It can lyse erythrocytes and a variety of other cells including mast cells, neutrophiles, and polymorphonuclear cells as well as enhance virulence by causing tissue damage or by dissolving material that would prevent spreading of the pathogen throughout the tissue. Several extracellular hemolysins have been characterized from *Vibrio* spp. The thermostable hemolysin from *V. parahaemolyticus* exhibited enterotoxic effects on human and rat cell monolayers [73,74]. To date, *Vibrio* hemolysin genes have been isolated from *V. cholerae* [75], *V. parahaemolyticus* [76], *V. anguillarum* [77] and *V. mimicus* [78]. However, the role and biological properties of hemolysin from *V. fluvialis* have been studied by Han *et al.* [79]. They found that hemolysin of *Vibrio fluvialis* (VFH) forms pores in erythrocyte membrane and by using osmotic protectants. The authors estimate the diameter of the pores to be 2.8–3.7 nm. This size seems larger than those formed by other *Vibrio* hemolysins such as *V. cholera* [80,81], *V. parahaemolyticus* [82], *V. metschnikovii* [83], *V. mimicus* [84], and *V. vulnificus* [85]. They suggested that VFH, a major hemolysin of *V. fluvialis*, is a pore forming toxin and induces osmotic lysis in erythrocytes. Chakraborty *et al.* [86] report that *V. fluvialis* showing cytotoxic and vacuolating activity on HeLa cells. These strains are also capable of causing haemolysis on sheep red blood cells. The utilization of heme compounds by *V. fluvialis*, although an iron acquisition system mediated by the catecholate siderophore fluvibactin has been reported [87]. Expression of iron-regulated proteins in *Vibrio* spp. has been related to increased virulence in animal models, but the role of heme utilization proteins in bacterial survival under oxidative stress and their effect on the production of pathogenic factors such as hemolysin is unknown. Ahn *et al.* [88] report the identification of the heme utilization protein HupO, which mediates the acquisition of iron from hemin in *V. fluvialis* and has amino acid sequence homology to bacterial outer membrane heme receptors.

## 8. Antimicrobial Resistance

An increase in the emergence of multi-antibiotics resistant bacteria in recent years is worrisome and the presence of antibiotics resistance genes on bacterial plasmids has further helped in the transmission and spread of drug resistance among pathogenic bacteria [89]. Antimicrobial resistance has become a major medical and public health problem as it has direct link with disease management [90]. Antibiotics such as tetracycline, doxycycline, norfloxacin, ciprofloxacin, streptomycin and fluoroquinolones may be used as an adjunct to rehydration therapy and are critical in the treatment of septicemia patient [91,92].

Multiple antibiotics resistance gene cluster with the same genetic locus (Resistance Island) can be transferred to other organisms. Spread of antibiotic resistance in microbes has been attributed to the mobilization of drug resistance marker by a variety of agent like plasmid, transposons, integrons and SXT element [93,94]. Ahmed *et al.* [95] reported that *V. fluvialis* have showed typical multidrug resistance phenotypes of SXT. In their findings, they observed that *V. fluvialis* was resistant to chloramphenicol, streptomycin, cotrimoxazole (trimethoprim and sulfamethoxazole), ampicillin, furazolidone, nalidixic acid, and gentamicin, and concur with our recently conducted research [96]. There are few factors that may contribute to the *Vibrio* species antibiotic resistant. Firstly, a mutation in cellular DNA could modify the antibiotics target site or transport mechanism, causing a decreased action of the antibiotic on the cell [97,89]. Other factors were an extra gene product target site or transport mechanism [98].

## 9. Treatment

An effective treatment of diarrhoeal disease has the potential to substantially lower morbidity and mortality. The reduction of mortality from diarrhoeal is primarily related to the effective management of dehydration. In general, oral rehydration plus bismuth subsalicylate or loperamide is adequate therapy for mild to moderate diarrhoea (less than four stools per day) [99]. The gastroenteritis syndrome is usually self-limited and does not require parenteral therapy [25,36], while the sepsis syndrome and the cellulitis syndromes are potentially life and limb threatening and require aggressive antibiotics therapy. Any delay in treatment in the later two syndromes increase the likelihood of poor outcomes for the patient especially if hypotension ensues. There is debate over which antibiotic regime is most effective given to multiple resistance pattern of *V. fluvialis* pathogen. Haq and Dayal [100], recommend 100 mg doxycycline intravenously every twelve hours, combined with two grams ceftazidime intravenously every eight hours. A group in Taiwan performed *in vitro* antibacterial testing and found several cephalosporin antibiotics effective in killing *Vibrio* infections including ceftazidime, ceftriaxone and cefotaxime [92]. They also found imipenem and a variety of quinolones to be equally efficacious. Several prophylactic and treatment drug regimes have been described for *Vibrio* infections with quinolones being the current drugs of choice for both prophylaxis and treatment [92]. Yet, the use of quinolones in the pediatric population remains controversial. The combined therapy with doxycycline and ceftazidime is the recommended according to CDC website. For treatment of children in whom doxycycline is contraindicated, the CDC website recommends a combination of trimethoprim-sulfamethoxazole and aminoglycoside [101].

The initial antibiotic choice for bacterial peritonitis often is empiric, based on the most likely pathogens, and the 3^rd^ generation cephalosporin or ampicillin plus aminoglycoside are commonly used. Third-generation cephalosporins, doxycycline, amoxicillin / clavulanate, and fluoroquinolones were commonly used for extra-intestinal *Vibrio* infections, mainly with *V. vulnificus* [37]. The treatment guideline for extra-intestinal *V. fluvialis* infection are not established, and the antibiotics were different in each case: cefuroxime and trimethoprim/sulfamethoxazole, gentamicin and ciprofloxacin, ceftazidime and oxytetracycline [37–39]. Further survey is needed to decide the appropriate treatment of choice for extra-intestinal infection by *V. fluvialis*.

## 10. Prevention

### Public Health Implication

The prevention of the spread of *V. fluvialis* strain cholera-like diarrhoea depends on ensuring appropriate sanitary measure like hand-washing, proper food preparation, efficient sewage treatment system disposal. Proper surveillance of water, food, and sanitation facilities, using public health diagnostic and detection procedures is necessary to protect individuals including infants from this cholera-like diarrhoea.

Environmental health protection measures that can be applied in agricultural use of wastewater for irrigation including wastewater treatment, crop restriction, control of wastewater application and human exposure, and promotion of hygiene [102], since consumers of irrigated crops that are likely to be eaten uncooked are at high risk for direct contact with pathogens leading to this disease. As with drinking water quality surveillance, finding affordable ways of monitoring the presence of harmful contaminants in wastewater that can accrue in soil and crops is essential.

In evaluating the degree of the *V. fluvialis* risk attention should be directed to control of fish and shellfish at the point of capture/harvest, a good sanitary management of farms and new regulatory initiative should be adopted both to limit losses caused by emerging pathogens and avoid the transmission of foodborne diseases to consumers, and also consumers should be informed of the risk they incur in eating raw or undercooked fish and seafood because, even when all preventive measure have been applied, pathogenic bacteria may have accumulated in the live animals.

The essential step to protect against emerging diseases caused by *V. fluvialis* is an effective global surveillance system to give early warning of infections. Medical practitioners must be aware of *V. fluvialis* infections and contribute to the identification of the risk factor associated with these pathogens in order to suggest specific preventive measures.

## 11. Conclusion

A number of circumstances could influence the emergence and re-emergence of different *Vibrio* species as significant pathogens in both developing and developed countries. *V. fluvialis* ranks very high as a human public health hazard amongst bacterial pathogens as well as a contaminant in marine foods and food products, and causing impairment in both freshwater and marine environments. The prime challenge in preventing the spread of these pathogens is poverty, which goes with poor sanitization, which has always been a bane of developing nations. There is need for proper surveillance of water, food and sanitation facilities to eradicate *V. fluvialis* malaise.
